# The palliative care needs and experiences of people with advanced head and neck cancer: A scoping review

**DOI:** 10.1177/0269216320963892

**Published:** 2020-10-21

**Authors:** Catriona R Mayland, Qiaoling Marilyn Ho, Hannah C Doughty, Simon N Rogers, Prithvi Peddinti, Praytush Chada, Stephen Mason, Matthew Cooper, Paola Dey

**Affiliations:** 1 Department of Oncology and Metabolism, University of Sheffield, Sheffield, UK; 2 Palliative Care Institute, University of Liverpool, Liverpool, UK; 3 Sheffield Teaching Hospitals NHS Foundation Trust, Sheffield, UK; 4 Nanyang Technology University, Singapore; 5 Department of Primary Care and Mental Health, Institute of Population Health, University of Liverpool, Liverpool, UK; 6 Faculty of Health, Social Care and Medicine, Edge Hill University, Ormskirk, UK; 7 Aintree University Hospitals NHS Foundation Trust, Liverpool, UK; 8 University of Liverpool Medical School, Liverpool, UK; 9 Liverpool University Hospitals NHS Foundation Trust, Liverpool, UK; 10 Luton and Dunstable University Hospital, Luton, UK

**Keywords:** Head and neck cancer, palliative care, palliative medicine, end-of-life, healthcare services, review

## Abstract

**Background:**

The palliative care needs of people with advanced head and neck cancer pose unique complexities due to the impact the illness has on eating, speaking, appearance and breathing. Examining these needs would help provide guidance about developing relevant models of care and identify gaps in research knowledge.

**Aim:**

To identify and map out the palliative care needs and experiences for people with advanced head and neck cancer.

**Design:**

A scoping literature review following the methods described by the Joanna Briggs Institute.

**Data sources:**

An electronic search of the literature was undertaken in MEDLINE (Ovid), EMBASE and CINAHL covering the years January 1996 to January 2019.

**Results:**

People with advanced head and neck cancer often had palliative care needs but there was variability in the timing and access to relevant services. A high prevalence of interventions, for example hospital admissions were needed even during the last month of life. This was not necessarily negated with early engagement of palliative care. Dissonance between patients and family carers about information needs and decision-making was an additional complexity. Studies tended to be descriptive in nature, and often involved a single centre.

**Conclusion:**

This scoping review demonstrates the complexity of care for people with advanced head and neck cancer and the issues related to the current healthcare systems. Focus on appropriate referral criteria, increased integration and coordination of care and robust evaluation of specific care components seems key. Linkage between research and service design delivery across teams, disciplines and care settings seems pertinent.

**What is already known about the topic?**

- Advanced head and neck cancer patients have specific challenges due to the impact of the illness on vital functions such as eating, speaking and breathing.
- Identifying the palliative care needs of this specific cancer subgroup would help provide guidance about how services could best provide care.

**What this paper adds?**

- Advanced head and neck cancer patients have a diverse range of palliative care needs, but there is variability in terms of access and timing to palliative care services.
- Dissonance between patients and family carers about information needs and decision-making represent additional complexities.
- Head and neck cancer patients frequently require acute interventions even during the last weeks of their life.

**Implications for practice, theory or policy**

- Tailored needs-based referral systems for advanced head and neck cancer patients may help address issues relating to access to palliative care services.
- Models of care focused on increased integration and coordination across different care settings and multi-disciplinary teams may help address issues relating to frequent use of acute interventions during the last weeks of life.
- Prospective multi-centre studies, potentially using mixed methods approaches, and focused on testing specific components of care may help further understand and tailor services more appropriately to meet needs.

## Introduction

On a global scale, head and neck cancer is the sixth most common cancer,^1^ representing a wide-ranging group of cancers arising from the epithelial lining of the upper aerodigestive track, and affecting the oral cavity including the lips; pharynx; larynx; paranasal sinuses and nasal cavity; salivary glands and middle ear. Within certain parts of the world, for example the United Kingdom (UK), the incidence of head and neck cancer is expected to rise by 50% over the next 20 years. This trend is attributed to more cases caused by Human Papilloma Virus (HPV).^2^ Although there is variability depending on the underlying histology, the overall 5-year survival rate has remained at 40% to 65%,^3^ due to factors such as advanced stage disease presentation and co-morbidities. Additionally, one in every five people with head and neck cancer will die within 12 months following diagnosis.^4^ Worldwide, poverty and socio-economic deprivation impact on survival, raising concerns about inequalities or disparities in access to healthcare services, including palliative care provision.^5,6^

Compared with other cancers, the palliative care needs of people with advanced head and neck cancer pose unique complexities due to the impact the illness has on eating, speaking and breathing.^7–9^ Head and neck cancer can be very visible, often causing facial disfigurement^10^ and distorted or unintelligible speech. Patients have distinct care challenges and can require feeding tubes and tracheostomies to support their vital functions. Symptoms can cause significant psychological distress and social isolation and there is a higher risk of suicide compared with the general cancer population.^11^ Earlier in the disease trajectory, family carers to those with head and neck cancer also report distress and unmet needs.^12,13^

Given the overall prognosis and potential for rapid demise for a significant proportion of head and neck cancer patients, it is important to consider support at the time of diagnosis for those with advanced disease. Understanding the palliative care needs and experiences of this vulnerable population is important to help devise relevant models of care and identify future research gaps. Although there have been two systematic reviews on unmet needs for advanced cancer patients,^14,15^ none have specifically focused on people with advanced head and neck cancer. Head and neck cancer brings unique challenges due to the anatomical location of the illness, and the fact patients are likely to experience significant symptom (physical and psychological) and healthcare burden regardless of what treatment course is chosen.^16^ A scoping review is beneficial to examine broad areas and is particularly useful to report on the types of evidence which may inform practice or identify key gaps in the evidence.^17^ The aim of this scoping review was to examine and map the palliative care needs and experiences for people with advanced head and neck cancer. As the research objective was wide-ranging, and the identified study designs were expected to be heterogeneous, a scoping review approach was deemed more appropriate compared with systematic review methodology.

## Methods

### Literature review question

The specific question to be addressed was:

What types of palliative care needs and challenges have been reported by people with advanced head and neck cancer, their family carers, and the healthcare professionals looking after them, in terms of their experiences and usage of healthcare services?

### Design

The Joanna Briggs Institute Scoping Review framework was used to guide conduct of the review.^17^ This framework represents well-established, detailed guidance, has previously been used to assess the quality of scoping reviews,^18^ and helped, in part, frame the development of the PRISMA extension on the reporting of scoping reviews.^19^

### Search strategy

**Figure 1.**
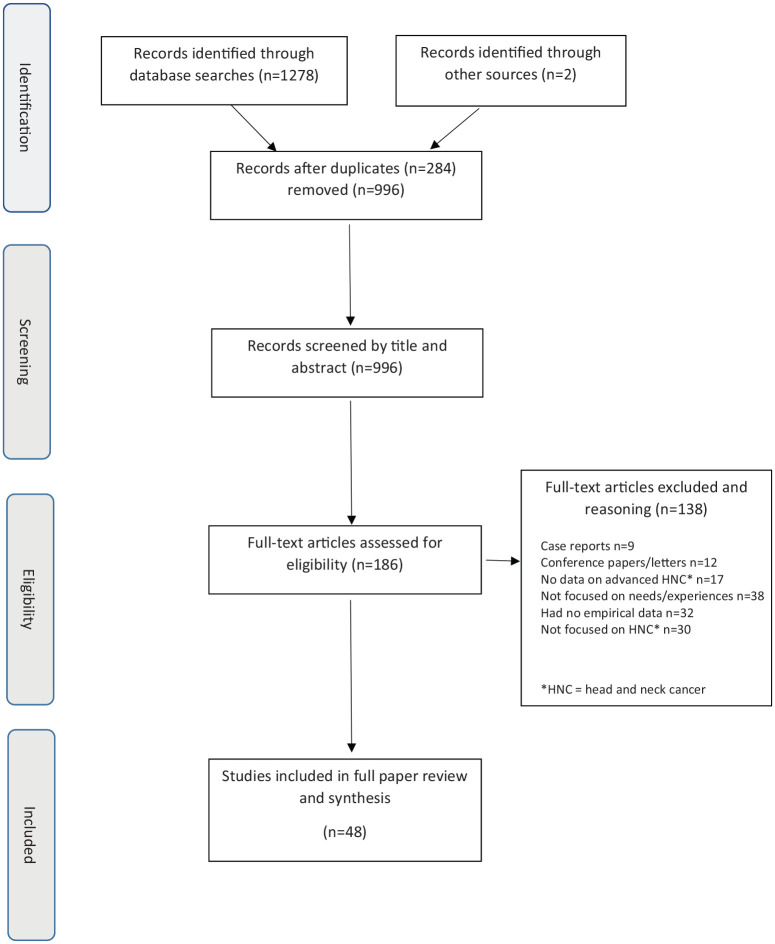
Flow diagram for the scoping review process.

An electronic search of the literature was undertaken in MEDLINE (Ovid), EMBASE and CINAHL covering the years January 1996 to January 2019. It was undertaken using keywords and subject heading terms for ‘Palliative care’ and ‘head and neck neoplasms’ (Textbox 1) using specified inclusion and exclusion criteria (Textbox 2). The searches were initially run on 12th April 2017 and further updated on 8th February 2019. The full MEDLINE search is available in Supplemental Table 1 and the other searches are available on request of the corresponding author. Titles and abstracts were initially screened (CRM, QMH or MC) to identify potentially eligible papers and any areas of uncertainty were resolved by another reviewer (PD). The full manuscripts of potentially eligible papers were further independently screened against eligibility criteria by two reviewers (either CRM and PP, CRM and PC, HCD and PD), with a third reviewer (PD or CRM dependent on the team) resolving any conflicts of opinion, to determine a definitive list of included studies (Figure 1). No additional hand searching was conducted but references of the included papers were also screened for any other relevant papers that might have been missed by the search.

**Textbox 1.** Search terms used for scoping literature review.

**Head and neck cancer** 1. exp Oropharyngeal Neoplasms/ 2. ‘Head and Neck Neoplasms’/ 3. exp Otorhinolaryngologic Neoplasms/ 4. exp Neoplasms/ 5. (cancer* or carcinoma* or neoplas* or tumor* or tumour* or malignan* or SCC).tw. 6. 4 or 5 7. exp Oropharynx/ 8. (oropharyn* or mesopharyn* or tonsil* or (head adj3 neck) or ‘tongue base’).tw. 9. 7 or 8 10. 6 and 9 11. (HNSCC or SCCHN or OP-SCC or OPSCC).tw. 12. 1 or 2 or 3 or 10 or 11 Palliative care 13. exp Palliative Care/ 14. exp Terminal Care/ 15. exp Terminally Ill/ 16. palliat*.mp. [mp=title, original title, abstract, name of substance word, subject heading word, unique identifier] 17. 1 or 2 or 3 or 4 18. 12 and 17

**Textbox 2.** Inclusion and exclusion criteria.

**Inclusion criteria** • Empirical research studies (any research design) of palliative care needs and experiences • Involving people with advanced head and neck cancer, or family carers of adults with advanced head and neck cancer, or healthcare professionals supporting adults with advanced head and neck cancer • Within any healthcare setting and any country • Published in English *Advanced head and neck cancer defined as involving those with incurable disease and/or being treated with palliative intent **Exclusion criteria** • Any studies where the primary focus involves children (⩽18 years old); patients who have solely undergone curative treatment; or survivorship issues • Case reports, case series, opinion pieces or letters

### Data extraction

Data was extracted using a specially designed proforma by one member of the research team (CRM, PP, PC or PD) and 75% were checked by a second reviewer (CRM or PD). Data was mapped out in a descriptive manner according to the following: setting, country, population characteristics, study design, intervention (where appropriate) and findings. The World Health Organisation’s definition of palliative care includes the ‘early identification and impeccable assessment and treatment of pain and other problems, physical, psychosocial and spiritual’.^20^ Therefore, prior to the search, the team had agreed key themes within the protocol based on the expert knowledge within the team, and anticipating the likely complexities relating to communication and need for interventions to support vital functions of relevance to people with head and neck cancer. These themes were: symptom control; psychological well-being; communication and/or decision-making; place of care and death and medical interventions at the end-of-life.

### Data analysis

Following the data extraction, the studies were categorised to one or more of these themes based on their findings, and the themes revised accordingly, following review and discussion by members of the team (CRM and PD). Following this, the data was tabulated and synthesised within each of the final themes. The final themes agreed were:

- overall palliative care need and access to palliative care services
- physical symptoms
- psychosocial and spiritual well-being
- medical interventions in the last 12 months of life
- communication and decision making
- place of death.

Due to the wide range of different study methodologies, and in keeping with the accepted remit of scoping review guidance, specific quality appraisal was not conducted.^17^ Instead, key study limitations, where documented within the manuscripts, were extracted to inform the synthesis of data within themes.

## Results

### Range of studies

From 1278 initial records, 185 papers were screened for eligibility, of which 46 were included in the full review. An overview of the characteristics of these 46 included studies is provided in Supplemental Table 1.

Studies were most commonly conducted in Europe (*n* = 23)^8,22,23,29,31–33,35,37,43–45,47–49,51,52,55,57,59,63–65^ with 12 being conducted in Asia,^24–28,34,40,42,45,53,61,62^ nine in North America,^30,36,38,39,41,50,54,57,58^one in Saudi Arabia^21^ and one in Australia.^60^ Quantitative methods were used for the majority of the studies (*n* = 41) with four using qualitative methods^29,36,52,65^ and one study utilising mixed methods.^59^ All the quantitative studies were descriptive or observational in nature (cross-sectional survey, case-control, or cohort studies). Ten studies were nation-wide studies^25–27,30,37,40,47,50,61,62,65^ and four were based within a specific region.^39,49,64^ The remaining 32 studies were conducted within single institutions. Thirty-six studies focused purely on patients,^21–28,30–41,45–50,53–55,57,59–64^ two on family carers alone,^44,58^ and two on healthcare professionals alone.^43,65^ A further four focused on both patients and family carers,^8,42,51,52^ and two on patients and healthcare professionals.^29,56^

For each key area, studies are presented in Tables 1 to 6; studies which have findings about more than one key area are reported separately within the different tables.

**Table 1.** Overall palliative care needs and access to palliative care services for advanced head and neck cancer patients.

Study	Aim	Main findings	Reported limitations
Overall palliative care needs			
Becker et al.^23^	Identify percentage of cancer patients with palliative care needs (PCN)	PCN prevalence highest in HNC patients (135/477, 28.3%) compared with other cancer groups (as defined by WHO definition and assessed by treating physician)	Single-centre analysisChallenging to define PCN
Rylands et al.^57^	Report treatment selection, survival, health-related quality of life, cause and place of death in relation to deprivation status	246/523 (47%) resided in the ‘most deprived’ IMD quartile37/67 (55%) of those receiving palliative treatments lived in ‘most deprived’ IMD quartileThose in more deprived areas reported more social-emotional dysfunction and worse overall quality of life (levels of physical functioning were similar)	Not specifically reported
Shah et al.^60^	Estimate frequency of referral of HNC patients to ‘terminal care’Ascertain where and when the patient died	74 (18%) patients were referred for palliative managementMean survival 5.5 monthsOverall 1-year survival = 11% of the cohort (*n* = 8 patients)	Small numbersShort durationNo details about specifics of palliative care/hospice care received
Timon and Reilly^63^	Assess group of incurable HNC patients presenting for the first time to one surgeonEmphasis on natural history and palliative therapy required	60/286 patients (21%) deemed appropriate for palliative care following initial diagnosisReason included advanced disease (*n* = 39), poor medical condition (*n* = 13) and patient refusal of curative treatment (*n* = 8)Palliative treatment included operative interventions (*n* = 22, 37%), tracheostomy (*n* = 10), PEG insertion (*n* = 14); 26 patients (43%) had palliative radiotherapy	Not specifically reported
Access to palliative care services			
Enomoto et al.^30^	Compare monthly Medicare costs for all services used during last 12 months of life by HNC patients – comparing those who received hospice care with those who did not	Most patients were enrolled in hospice care within 30 days of death (63.4% for oral cancer; 57.8% for pharyngeal cancer).Mean cost for patients enrolled in hospice was >$7000 less compared with those who weren’tFemale gender, white race, being married and increasing year of diagnosis were significantly associated with higher hospice use	Other health outcomes not included for example, QoL, patient satisfactionDidn’t include unpaid carer costs
Hui et al.^38^	Determine proportion and predictors of cancer patients who receive palliative care (PC)	Multi-variate analysis showed older age, being married, and specific cancer types for example gynaecological, lung, HNC were significantly associated with PC referral (although OR 1.01 for HNC, 95% CI 0.53–1.96)Time between PC consultation and death was 2 months for HNC patients	Retrospective natureDidn’t include clinical outcomes, for example, QoLPatient may have been offered but declined PC consult
Johnstone et al.^39^	Assess whether Nova Scotia cancer patients (who may need palliative care) are being referred to the comprehensive Halifax-based Palliative Care Program (PCP)	PCP referral was more likely for those who died with HNC (OR 5.4, 95% CI 3.0–9.7)HNC was less likely to be a predictor of late PCP referral	Not specifically reported
Kwon et al.^41^	Define characteristics, outcomes and utilisation of medical services by cancer patients referred early in their disease course to outpatient palliative care services	Early referrals (ERs) = those who were receiving or had completed treatment with curative intent or had an expected survival time of more than 2 years. From all referrals, 73/200 (37%) had HNCThe most common tumour type for ERs was HNC (67%)Logistic regression analysis showed having HNC was an independent predictor for early referral to palliative care service (OR 9.5, CI 3.09–27.08, *p* < 0.0001)	Population selected based on first referral to Supportive Care Centre rather than prognosis/symptom distress
Mulvey et al.^50^	Determine incidence of palliative care consultations (PCC) among hospitalised metastatic, incurable HNC patientsExamine relationship between palliative care encounters and in-hospital morbidity, mortality, length of hospitalisation and costs	PCC was documented in 4029 cases (5%)PCC significantly associated with age >80 years; female gender; self-pay payor status (uninsured); and prior radiationPCC significantly less likely for those with Medicare/Medicaid; receiving chemotherapy (OR 0.27, *p* < 0.001) or radiation (OR 0.60; *p* = 0.037) during admissionPCC associated with reduced hospital-related costs but not length of hospital stay	Analysis of long-term outcomes not possibleCertain costs for example, physician-related costs not included in databaseUnable to determine if PCC was provided by certified palliative care physician
Tang et al.^62^	Evaluate associations between hospice utilisation in the last year of life and patient demographics, disease characteristics, physician specialty, hospital characteristics and availability of healthcare resources at the hospital and regional levels in Taiwan	HNC patients (along with those with breast, liver, pancreatic or bile duct and gastric cancer) more likely to use hospice services compared with lung cancer patients (OR 1.06, 95% CI 1.01–1.10)	Observational study – potential impact of unmeasured factors for example, patient attitudes towards end-of-life care
Ullgren et al.^64^	Describe HNC patients referred to palliative care and how care transition from acute oncological to palliative care impacted on Health Related Quality of Life (HRQoL) and informationExplore HNC patients’ HRQoL and perceived information	43/202 (21%) patients had been referred to palliative careThose referred were more likely to have undergone multi-modal treatment; have lower levels of global health and higher symptom burden (fatigue, pain, nausea, vomiting) compared with those without palliative care referralThose referred reported they had visited ED more frequently compared to group without palliative care (18/43, 43% vs 22/114, 19%)Those referred to palliative care had lower levels of perceived information about causes of disease, extent/spread of disease compared with those without palliative care referral	Small proportion referred to palliative careDidn’t use HNC specific instruments

CI: confidence intervals; ED: emergency department; HNC: head and neck cancer; IMD: index of multiple deprivation; OR: odds ratio; PEG: percutaneous endoscopic gastrostomy; QoL: quality of life; WHO: World Health Organisation.

**Table 2.** Physical symptoms in advanced head and neck cancer patients.

Study	Aim	Main findings	Reported limitations
Physical symptoms in advanced head and neck cancer			
Alt-Epping et al.^22^	Assess symptoms and psychosocial needs of patients with incurable HNC	Mean QoL 87.7/148 for 22 patients – using Functional Assessment of Chronic Illness Therapy Head and Neck Module (FACT-H&N), 0 = worst, 148 = best QoLBeing unable to eat the favoured foods; being able to eat solid foods; suffering from a dry mouth were almost uniformly present	Small numbersQuestionnaires not fully understood by participants
Bisht et al.^24^	Examine effect of palliative drug therapy on QoL in advanced HNC patients	Pain most frequent symptom (in 38/40, 95% patients); frequent polypharmacotherapy (mean 8.7 drugs)Improvement in QoL – baseline mean score 950.39 versus 1336.67 at 1 month and 1405.49 at 2 months (range 0–2800) after using palliative drug therapy	Small numbersShort duration of follow-upQoL measure not specific for HNC
Forbes^32^	Outline nature, incidence and management of problems and the role of the hospice in the patients care	Patients had a median of 6 symptoms (range 2–12)28 (74%) patients had dysphagia and difficulty feeding30 (79%) had weight loss or pain; strong opioids were prescribed for 13 (34%)18 (47%) had bleeding from a wound and/or tracheostomy20 (53%) had a tracheostomy, 8 had a NGT and 1 had a PEG	Not specifically reported
Gupta et al.^34^	Measure QoL in upper aerodigestive tract cancer (UADT) cancer patients in comparison with hospital controls	Mean composite QoL score for cases was poorer at 62.85/100 compared with 89.14/100 for controls; patients with oropharynx and hypopharynx cancer had worst mean scores across all domainsStage IV disease had worst mean score for pain, appearance, activity and recreationStage III disease had worst mean score for swallowing, chewing, speech, mood and anxiety	Not specifically reported
Heinonen et al.^35^	Describe current status of palliative care of HNC patients in one specific university hospital region	From 60 HNC patients, 45% had a PEG and 28% had a tracheostomy98% received opioids during palliative phase; 77% laxatives; 43% anti-emetics; 40% benzodiazepines	Small numbersRetrospective natureSymptoms not systematically reported
Lal et al.^42^	Evaluate range of symptoms, other needs and evaluation of treatment strategies, especially for pain management	Pain most common symptom (134/153, 87%), then fungating lesion (47/153, 31%) and difficult/painful swallowing (61/153, 40%)Median duration of symptoms was 4 months (range 1–48 months)103/134 (77%) had mixed nociceptive and neuropathic pain; 95/134 (71%) had moderate/severe pain and needed opioids101/134 (65%) needed NGT feeding; 80 (52%) had on-going weight loss	Retrospective nature
Ledeboer et al.^43^	Evaluate experience of GPs in the care of palliative HNC patients, experiences of communication and consultation of attending specialists	Only 45% GPs perceived their patients were satisfied with their symptom control	Retrospective natureViews of GPs (rather than patients)
Lidstone et al.^45^	Assess prevalence and severity of symptoms and concerns – identify patient groups who might benefit from routine SPC involvement in outpatient clinics	HNC patients reported highest prevalence of mouth and taste problems (38/60, 63%), and swallowing problems (30/60, 50%)31/60 (52%) HNC patients had pain and 26/60 (43%) reported change in appetite46/60 (77%) had a lack energy	Not specifically reported
Lin et al.^46^	Describe symptom patterns of terminal HNC patients in palliative care unit	Most common symptom was weight loss (97.9%); then pain (96.8%), cough (95.7%), dysphagia (90.4%), feeding difﬁculties (89.4%) and communication difﬁculties (78.7%)33/94 patients had tracheostomyMedian equivalent morphine dose at admission was 70 mg/day (range 0–1080) and 160 mg/day (range 0–1600) immediately prior to death	Not specifically reported
Lokker et al.^8^	Determine prevalence and impact of symptoms on daily functioning in HNC patients during palliative phaseExamine discrepancies between patients and family members symptom scoring	HNC patients reported an average of 14 symptoms (range 0–26) of which 10 were somatic symptomsFatigue had highest prevalence (81%), then pain (75%), weakness (75%), trouble with short walks outside (65%) and dysphagia (59%).Dyspnoea, voice changes, trouble with short walks outside and weakness had greatest impact on daily functioningPerceptions about symptom impact on daily functioning differed between patients and their family members for ‘trouble with short walks’ and ‘difficulty sleeping’	Didn’t use validated questionnaires44% non-response rateLimited numbers
Mercadante^48^	Establish degree of opioid sensitivity and possible factors involved in advanced stage HNC patients being followed up at home	Patients had mixed pain syndromes (23 somatic, 19 visceral, 19 neuropathic)12/37 patients had good and 25/27 partial responsiveness to opioid14/37 had steroids, 14/37 had anti-inflammatory drugs; amitriptyline and carbamazepine administered > 2 weeks in 8 and 11 patients respectively13/47 patients had subcutaneous morphine	Not specifically reported
O’Sullivan and Higginson^52^	Explore Irish HNC patient and care-givers views on EoL care	Concerns about symptom control perceived as a potential barrier to dying at home	Some patients were in remission so views may alter as disease progresses
Price et al.^54^	Understand cause and location of death and symptoms experienced at the end of life	89/93 (94.7%) patients had at least one symptom in the 6 months prior to deathMost common symptom was pain, then dysphagia, anorexia/weight loss, fatigue/weakness and dyspnoea (mean of 4.7 symptoms per patient)	Retrospective natureSmall number
Roscoe et al.^56^	Understand ways in which end-stage HNC patients and their oncologists talk about end-of-life issues	Patients overall QoL rated 7.29 (SD 2.81) on 0–10 scale (10 = excellent)Most prevalent symptoms were pain, constipation, inability to use their mouth, dry mouth, mucus, weakness, fatigue, shortness of breath, anxiety, insomnia and speech problems	Small numbersSingle assessment rather than longitudinal
Shuman et al.^58^	Determine perceived quality of care for HNC patients at the end of their lives	Mean score for ‘management of symptoms’ = 31/100 (lowest mean score from all 9 assessed domains) as perceived by 58 bereaved family members	Poor response rateRetrospective natureDifferent environment for using validated tool
Shinozaki et al.^61^	Examine relationship between QoL and functional status in terminally ill HNC patients	No significant change in QoL between baseline and week 3 (using the European Organization for Research and Treatment of Cancer Quality of Life Questionnaire (EORTC QLQ)-Core 15-Palliative Care (C15-PAL)32 (44%) had a ‘tracheostoma’; 53 (74%) had enteral feedingMedian duration of hospital stay shorter for PEG-fed patients (21 days) compared with NGT-fed patients (64 days)Fungating tumours requiring dressing changes in 22 (31%) patients; 5 (7%) patients had severe bleeding – 2 were fatal (2.8%)	Small numbersLimited to in-patients only
Physical symptoms specifically in last 1–2 weeks of life			
Ethunandan et al.^31^	Evaluate quality of dying experience by examining symptoms in the last week of life	27/32 (84%) had pain in week preceding death; 25/27 received opioids (18 given parentally)20/32 had difficulty swallowing – 6 had a PEG and 1 had NGT (none inserted in last week life). 11 had urinary incontinence and 3 had faecal incontinenceBleeding was an issue in 5 patients	Retrospective nature
Fullarton et al^33^	Record characteristics, mode of death and potential indicators of the quality of care at the end of life for HNC patients	In last week of life, 33/76 (43%) patients had pain; 20/76 (26%) had dyspnoeaPrevalence of pain was higher in hospice (12/13, 92%) and was main reason for admission	Retrospective natureMissing dataHeterogeneous groupLimited number of hospice patients
Sesterhenn et al.^59^	Describe end-stage disease in advanced HNC patients – circumstances of final period of life and describe period in hospice setting	Intensive nursing support needed in hospice – 11/16 patients had tracheostomy; 13/16 received CAN (10 = PEG, 3 = NGT)7/16 were incontinent rising to 12/16 in the two weeks prior to death	Not specifically reported

CAN: clinically-assisted nutrition; EoL: end of life; GPs: general practitioners; HNC: head and neck cancer; NGT: nasogastric tube; NOK: next-of-kin; PEG: percutaneous endoscopic gastrostomy; QoL: quality of life; SD: standard deviation; SPC: specialist palliative care.

**Table 3.** Psychosocial and spiritual well-being in advanced head and neck cancer patients.

Study	Aim	Main findings	Reported limitations
Psychosocial and spiritual well-being in advanced head and neck cancer			
Alt-Epping et al.^22^	Assess symptoms and psychosocial needs of patients with incurable HNC	12/22 (54.5%) patients reported distress levels of ⩾7/10 (using National Comprehensive Cancer Network distress thermometer)Elevated level of depression and anxiety found in half the patients (6 and 5 patients, respectively)	Small numbers (*n* = 22)Questionnaires not fully understood by participants
Gupta et al.^34^	Measure QoL in upper aerodigestive tract cancer (UADT) cancer patients in comparison with hospital controlsAssess impact of clinical predictors at time of diagnosis on QoL (using the University of Washington QoL questionnaire)	Most affected QoL domain was anxiety (cases mean score 21.6 versus controls 71.7, 0–100 scale, where 100 – best symptom/function), followed by mood (cases mean score 22.3 vs controls 63.0, 0–100 scale)	Not specifically reported
Henry et al.^37^	Understand lived experience of disfigurement in HNC and explore what patients considered to be its influences	Main theme of a ‘ruptured self-image – a discontinuity in one’s sense of self’ (‘I am no longer the same person’)Oscillation between this and search for normality to help reach acceptanceInvolves functional and lifestyle changes, existential components (living with a life-threatening disease) and has social implications	Selection bias – those who were comfortable speaking about topic, who were selected by healthcare professional; only those in urban hospital setting
Lal et al.^42^	Evaluate range of symptoms, other needs and evaluation of treatment strategies, especially for pain management	53/153 (35%) patients perceived (by oncologist assessment) to have some level of depression32/63 (46%) NOK perceived patient had a ‘peaceful’ death (low symptom burden, low level of psychological distress)	Retrospective nature
Ledeboer et al.^43^	Evaluate experience of GPs in the care of palliative HNC patients, experiences of communication and consultation of attending specialists	25/41 (61%) GPs perceived their patients had sufficient psychosocial care with 5% perceiving it as insufficient	Retrospective nature
Ledeboer et al.^44^	Increase knowledge of how treatment and support are experienced by relatives of palliative HNC patients during the palliative stage and after death	From 45 relatives, 67% reported the patients were ‘sometimes’ or ‘often’ depressed; 69% perceived patients needed better psychosocial support during palliative stageOnly 23% relatives reported there was spiritual support	Relatives feedback may not reflect actual patients’ perspective; time between death and questionnaire completion was a year; didn’t explore all head and neck specific issues
Lidstone et al.^45^	Assess prevalence and severity of symptoms and concerns – identify patient groups who might benefit from routine SPC involvement in outpatient clinics	From the 60 HNC patients, 31 (52%) had concerns about the future; 35 (58%) felt tense/worried/fearful; 34 (57%) felt low in mood/depressed	Not specifically reported
Lokker et al.^8^	Determine prevalence and impact of symptoms on daily functioning in HNC patients during palliative phaseExamine discrepancies between patients and family members symptom scoring	HNC patients had an average of 4 psycho-social symptomsThe most frequently reported psycho-social symptoms were ‘worrying’ (61%), ‘sadness’ (57%), ‘tenseness’ (52%), ‘depressed mood’ (52%), ‘powerlessness’ (50%).Perceptions about the symptom impact on daily functioning differed between patients and family caregivers for ‘anxiety’, ‘expressing oneself’ and ‘powerlessness’	Didn’t use validated questionnaires44% non-response rateLimited numbers
Offerman et al.^51^	Evaluate interventions/impact of newly established ‘Expert Center’ on palliative HNC patients as perceived by bereaved relatives	After establishing an ‘Expert Centre’ for HNC care, bereaved relatives perceived improved psychosocial support offered (68% vs 51% satisfied with Head and Neck Department)	Retrospective designCan’t be certain improvements purely relate to Expert Centre Feedback from relatives subjective
O’Sullivan and Higginson^52^	Explore Irish HNC patient and care-givers views on EoL care	Participants very willing to discuss most aspects of EoL care (preferences for place of care and death, prognostication) with no signs of psychological distressConcerns about family carer burden perceived as a potential barrier to dying at home	Some patients were in remission so views may alter as disease progresses
Patil et al.^53^	Identify the incidence of distress in HNC patients who are starting palliative chemotherapy and the factors associated with it	From 200 HNC patients, over 50% reported depression, fears, nervousness, sadness, worriesBaseline median distress score 3/10; 89 (44.5%) had high distress ⩾4/10; 88 underwent clinician counselling – 52 (59.1%) had a reduction in distress score to <4	Single centre study; post-hoc analysis
Roscoe et al.^56^	Understand ways in which end-stage HNC patients and their oncologists talk about end-of-life issues	Patients average depression scores were low (2.29, SD = 2.61) as were ratings of sadness (2.64, SD = 2.62) (measured on 10-point scale where 0 = never/not at all and 10 = very depressed or sad all the time)	Small numbers (*n* = 14); patients only asked on one occasion
Schuman et al.^58^	Determine perceived quality of care for HNC patients at the end of their lives	Perceptions by 58 bereaved relatives (from 286) deemed mean score for ‘emotional and spiritual support’ = 70/100Mean score for ‘well-being and dignity’ = 69/100	Poor response rateRetrospective natureDifferent environment for using validated tool
Psychosocial and spiritual well-being specifically in last 1–2 weeks of life			
Ethunandan et al.^31^	Evaluate quality of dying experience by examining symptoms in the last week of life	In the last week of life, 14/32 (44%) patients exhibited restlessness and confusion	Retrospective nature
Fullarton et al.^33^	Record characteristics, mode of death and potential indicators of the quality of care at the end of life for HNC patients	In last week of life, 15/76 (20%) patients were agitated	Retrospective natureMissing dataHeterogeneous groupLimited number of hospice patients
Sesterhenn et al.^59^	Describe end-stage disease in advanced HNC patients – circumstances of final period of life and describe period in hospice setting	Wide range of different mental statuses described by healthcare team about the 16 patients – including ‘desperation’, suicidal episodes, confusion and agitation.	Not specifically reported

EoL: end-of-life; GPs: general practitioners; HNC: head and neck cancer; QoL: quality of life; SD: standard deviation; SPC: specialist palliative care.

**Table 4.** Medical interventions with advanced head and neck cancer patients in the last 12 months of life.

Study	Aim	Main findings	Reported limitations
Alsirafy et al.^21^	Determine prevalence of hypercalcaemia in advanced HNC patients in a palliative care settingAssess impact of hypercalcaemia on administrative data that may indicate poorer EoL care	Hypercalcaemic patients more likely to be referred to palliative care, while they were inpatients (*p* = 0.004) compared with non-hypercalcaemic patientsDuring the last 3 months of follow-up, hypercalcaemic patients more likely to be hospitalised for ⩾14 days (*p* = 0.01) and visit emergency room more than once (*p* = 0.04)	Not specifically reported
Chang et al.^25^	Investigated relationship between demographics, primary physician’s specialty, hospital characteristic and ‘aggressiveness’ of their EoL care for oral cancer patients	Six indicators of ‘aggressiveness’ of EoL care in the last month of life: use of chemotherapy; >1 ER visit; >1 hospital admission; >14 days hospitalisation; an ICU admission; death in acute care hospital96% of 5386 deceased patients had at least one indicator – mainly ER visits and ICU admissionHigh utilisation of chemotherapy, ER and ICU in more than 50% of patients during the last month of life	Potential misclassification errorsNo QoL evaluationLack of information about impact of hospitalisation/chemotherapy in reducing symptoms
Chen et al.^26^	Determine prevalence of CPR in Taiwanese cancer patients in the last month of life and association with patient and physical characteristics	12% of 17,040 HNC patients had CPR in last month of life; higher compared with rest of cancers (10.5%).	Observational study so potential confounders
Ethunandan et al.^31^	Evaluate quality of dying experience by examining symptoms in the last week of life	13/20 HNC patients who died in hospital had a DNR order in place; none underwent CPR or had an ICU admission17/32 (53%) needed emergency admissions in last month of life – most commonly due to bleeding; then pain, respiratory problems, swallowing problems, not coping and fractured leg	Retrospective nature
Fullarton et al.^33^	Record characteristics, mode of death and potential indicators of the quality of care at the end of life for HNC patients	Main reasons for last admission to surgical ward prior to death were: for operation, airway management, or cancer diagnosis; 13/63 died suddenly within hospital ward	Retrospective natureMissing dataHeterogeneous groupLimited number of hospice patients
Heinonen et al.^35^	Describe current status of palliative care of HNC patients in one specific university hospital region	Median survival 11 months; during this time, 37/60 patients (66%) attended emergency department ⩾1x (range 0–6); 21 (35%) were hospitalised (most common reason was infection)12/34 (35%) who were referred to the specialised palliative home care died at home versus 3/36 (12%) of those who weren’t supported	Small numbersRetrospective natureSymptoms not systematically reported
Henson et al.^37^	Identify socio-demographic and clinical factors associated with end-of-life emergency department (ED) visits	Multiple ED visits in last month of life associated with diagnosis of lung or HNC (AOR 1.67, 95% CI 1.4–2.0)	Issues relating to routinely collected data for example, quality of data coding
Ledeboer et al.^44^	Increase knowledge of how treatment and support are experienced by relatives of palliative HNC patients during the palliative stage and after death	10/20 responses from relatives perceived the medical treatment as ‘too intrusive’ for the patient	Relatives feedback may not reflect actual patients’ perspective; time between death and questionnaire completion was a year; didn’t explore all head and neck specific issues
Mercandante et al.^49^	Assess patient characteristics who were hospitalised in last days of life after being assisted by a home palliative care teamIdentify possible risk factors for hospitalisation	138/550 (25.1%) admitted to hospital, of which 20 had HNCLogistic regression analysis showed patients with HNC (OR 2.62, 95% CI 1.24–5.54, *p* = 0.01) and lung cancer more likely to die in hospital (but very small numbers)	Single centre – results not generalisable
Offerman et al.^51^	Evaluate interventions/impact of newly established ‘Expert Center’ on palliative HNC patients as perceived by bereaved relatives	Relatives perceived a reduction in satisfaction with medical treatment (77% vs 81%) and poorer perceptions about whether HNC department did ‘everything to make life for the patient as comfortable as possible’ (64% vs 75%)13% relatives perceived the patient had treatment against their wishes	Retrospective design Feedback from relatives subjective
O’Sullivan and Higginson^52^	Explore Irish HNC patient and care-givers views on EoL care	Patients found it challenging to discuss preferred focus of care and ACPAll tended to favour maximum medical interventions associating palliative care with ‘giving up’ or ‘losing the fight’Whereas family carers perceived quality of life should have more priority compared with quantity of life	Some patients were in remission so views may alter as disease progresses
Randen et al.^55^	Describe how palliative chemotherapy is prescribed at the end-of-life to patients	15/25 (60%) HNC patients had chemotherapyAverage of 82 days (median 46 days) between last administration of chemotherapy and death (for all cancer groups)	Not specifically reported
Schuman et al.^58^	Determine perceived quality of care for HNC patients at the end of their lives	SPC team involvement improved perceptions of care at the time of death (*p* < 0.001)Palliative anti-cancer treatments (radiotherapy ± chemotherapy) improved perceptions about managing symptoms and care at the time of death (*p* = 0.011, *p* = 0.017).	Poor response rateRetrospective natureDifferent environment for using validated tool
Ullgren et al.^64^	Describe HNC patients referred to palliative care and how care transition from acute oncological to palliative care impacted on Health Related Quality of Life (HRQoL) and informationExplore HNC patients’ HRQoL and perceived information	43/202 (21%) HNC patients were referred to palliative carePalliative care group reported more frequent ER attendance compared with group without palliative care (18/43, 43% vs 22/114, 19%)	Small proportion referred to PCDidn’t use HNC specific instruments

ACP: advance care planning; AOR: adjusted odds ratio; CI: confidence intervals; CPR: cardio-pulmonary resuscitation; DNR: do not resuscitate; EoL: end-of-life; ER: emergency room; HNC: head and neck cancer; ICU: intensive care unit; OR: odds ratio; QoL: quality of life; SPC: specialist palliative care.

**Table 5.** Communication and decision-making issues in advanced head and neck cancer patients.

Study	Aim	Main findings	Reported limitations
Practical difficulties with communication			
Alt-Epping et al.^22^	Assess symptoms and psychosocial needs of patients with incurable HNC (at time of diagnosing incurability)	5/22 HNC patients depended on tracheostomyHad practical implications for conducting study – communication by telephone (e.g. for making appointments) not possible and needed face-to-face communication	Small numbersQuestionnaires not fully understood by participants
Forbes^32^	Outline nature, incidence and management of problems and the role of the hospice in the patients care	From 38 HNC patients, 20 (53%) reported communication as an issueFour patients could only communicate by writing	Not specifically reported
Lidstone et al.^45^	Assess prevalence and severity of symptoms and concerns – identify patient groups who might benefit from routine SPC involvement in outpatient clinics	From 460 patients, the 60 HNC patients reported the highest prevalence of problems with communication (30%) compared with other cancer groups	Not specifically reported
Lin et al.^46^	Describe symptom patterns of terminal HNC patients in palliative care unit	Statistically signiﬁcant association of communication difﬁculties with presence of a tracheostomy (*p* < 0.001)	Not specifically reported
Sesterhenn et al.^59^	Describe end-stage disease in advanced HNC patients – circumstances of final period of life and describe period in hospice setting	Communication difficult for most patients (due to tracheostomy, respiratory secretions or tumour obstruction); only 4/16 able to talk regularly and most had tracheostomy	Not specifically reported
Information provision and decision-making			
Chiu et al^28^	Identify most frequently encountered ethical dilemmas in the palliative care unit	HNC patients had more ethical dilemmas – 1.75/patient compared with other cancer sites (except gastric cancer)Dilemmas about place of care occurred most frequently with HNC patients (43.8%)Other frequent HNC patient dilemmas included problems accepting/complying with recommended discharge plans and dilemmas about hydration and nutrition	Not specifically reported
Dronkers et al.^29^	Investigate whether prognostic information on life expectancy is included during communication on diagnosis and treatment plans between physicians and HNC patients in all phases of illness	20/23 HNC patients received a curative treatment plan (*n* = 3, 13% palliative)Primary initiators of prognosis discussion – HNC surgeons 58%, patients 18%, caregivers 24%In 7 interviews, prognosis provided in quantitative manner that is, numerical probability estimates (13 quotations, 5.9%)In all 23 consultations, prognosis provided in a qualitative manner, that is, using words ‘most likely’ or highly improbable’ (*n* = 209, 94.1%)Two main communicative styles:Directive – including paternalistic language, use of medical jargonAffective – including patient-empowering professional attitude, giving hope, but also use of euphemisms	Bias due to patient participants being more engaged in this topic and physicians being aware their discussions are recorded
Ledeboer et al.^43^	Evaluate experience of GPs in the care of palliative HNC patients, experiences of communication and consultation of attending specialists	From 41 GPs, 33% weren’t informed by the hospital team the HNC was incurableProvision of information rated as 6.4 (on 0–10 point rating scale)Better communication associated with increased GP satisfaction with allocation of responsibilities (*p* < 0.01)54% GPs perceived information provided to patients was adequate; a fifth perceived important gaps	Retrospective nature
Ledeboer et a.^44^	Increase knowledge of how treatment and support are experienced by relatives of palliative HNC patients during the palliative stage and after death	33 (75%) relatives perceived the understandability of the information about the medical condition as ‘good’ or ‘very good’	Relatives feedback may not reflect actual patients’ perspective; time between death and questionnaire completion was a year; didn’t explore all head and neck specific issues
Offerman et al.^51^	Evaluate interventions/impact of newly established ‘Expert Center’ on palliative HNC patients as perceived by bereaved relatives	After establishing an ‘Expert Centre’ for HNC care, bereaved relatives’ perceived improved satisfaction about level of communication with surgeon (78% vs 59% perceived it as ‘good’ or ‘very good’)	Retrospective designCan’t be certain improvements purely relate to Expert Centre Feedback from relatives subjective
O’Sullivan and Higginson^52^	Explore Irish HNC patient and care-givers views on EoL care	In terms of communication/information, patients were divided between ‘full disclosure’ and a more ‘passive approach’Family carers favoured being fully informed to help them prepare and cope	Some patients were in remission so views may alter as disease progresses
Roscoe et al.^56^	Understand ways in which end-stage HNC patients and their oncologists talk about end-of-life issues	Patients’ mean score on general communication subscale from ‘Quality Of Communication’ (QOC) questionnaire was 8.47 (SD = 1.80) (10-point scale)Mean score on EoL subscale was 7.39 (SD = 2.82) indicating relatively high ratingsPatients, however, reported an absence of communication about key end-of-life topics; evidence of patients’ misunderstanding or misinterpreting information provided	Small numbers (*n* = 14); patients only asked on one occasion
Schuman et al.^58^	Determine perceived quality of care for HNC patients at the end of their lives	Mean score for ‘information and communication’ was 61/100 (SD 32.05)52/60 (90%) patients had advanced directives and 44 (76%) had DNR orders	Poor response rateRetrospective natureDifferent environment for using validated tool
Ullgren et al.^64^	Describe HNC patients referred to palliative care and how care transition from acute oncological to palliative care impacted on Health Related Quality of Life (HRQoL) and informationExplore HNC patients’ HRQoL and perceived information	From 289 patients, those referred to palliative care (*n* = 43) had lower levels of perceived information about causes of disease (*p* < 0.000) and extent/spread of disease (*p* < 0.001) compared with those without palliative care referral	Small proportion referred to palliative careDidn’t use HNC specific instruments
Xuereb et al.^65^	Explore local decision-making, from an ethical point of view, about HNC	Ten participants asked whether it was ethical to withhold treatment for low prognosis HNC patients (and only offer palliative treatment) – 3 agreed to withhold treatment and 7 considered this decision unethical (but emphasised informed consent should always take priority)8/10 participants agreed patients form an important part in the decision-making processFactors influencing level of patient engagement include intellect; co-morbidities; uncertainties about tolerance and outcome of treatment; avoiding false expectations	Not specifically reported

DNR: do not resuscitate; EoL: end of life; GPs: general practitioners; HNC: head and neck cancer; SD: standard deviation; SPC: specialist palliative care.

**Table 6.** Place of death for advanced head and neck cancer patients.

Study	Aim	Main findings	Reported limitations
Chen et al.^27^	Determine impact of patient demographics, disease characteristics, prognosis awareness and support network variables on preference for home death	More than half of 2881 participants expressed preference to die at home (*n* = 1,114, 54.7%)Having HNC was a factor significantly associated with preference to die at home (AOR = 1.57, 95% CI [1.10, 2.24], *p* = 0.012)	Convenience sample limiting generalisabilityDidn’t specifically look at hospice deaths (just home vs not home)
Fullarton et al.^33^	Record characteristics, mode of death and potential indicators of the quality of care at the end of life for HNC patients	From the 76 HNC patients, those dying in the hospice were younger compared with those who died in the hospital (mean age 63.7 (SD 11.0) years vs 70.6 (SD 11.9) years)	Retrospective natureMissing dataHeterogeneous groupLimited number of hospice patients
Kuo et al.^40^	Assess end-of-life care for patients with HNCs in Taiwan	From 98,221 HNC patients, those who were male, lived in more urbanised areas, had a higher family income, received hospice care in last month of life, had been prescribed opioids in last 3 months of disease were more likely to die at home or in hospice wardsThose who received chemotherapy, surgery or radiotherapy in last month of life tended to die during acute in-hospital admission (and total medical costs were higher)	Retrospective data analysisUnable to explore reasoning for decision-making
Lock and Higginson^47^	Describe the older population who die of cancer and the factors which may affect place of death	From 315,462 cancer deaths, hospice death was most common among those dying of HNC (19%)	Data completenessCross-sectional study so unable to control for potential confounders
Shah et al.^60^	Estimate frequency of referral of HNC patients to ‘terminal care’Ascertain where and when the patient died	Place of death identified for 51 HNC patients (from 74) – hospice (*n* = 22, 43%), home or nursing home (*n* = 13, 25%), hospital (*n* = 16, 31%)	Small numbersShort durationNo details about specifics of palliative care/hospice care received

AOR: adjusted odd ratio; HNC: head and neck cancer.

### Overall palliative care need and access to palliative care services

There were 11 studies reporting on these areas (Table 1). Studies suggest that 18% to 21% of all people with head and neck cancer received palliative management following diagnosis,^56,63^ with a higher rate seen for those residing in more deprived areas.^57^ There was some evidence that access may be lower than need, however, with one study estimating 28.3% of hospital in-patients had relevant needs.^23^

Head and neck cancer patients were more likely to receive palliative care or prompt a referral to palliative care services than other cancer patients^38,39,41,62^ due to the high degree of symptom burden.^64^ Timing of referral to palliative care teams varied, with two studies suggesting that head and neck cancer patients were referred early,^39,41^ and one study observing late referral, that is, in the last 30 days of life.^30^ Higher rates of access to palliative care services were observed among those with HNC who were older, white and female.^30,50^

### Patients’ physical symptoms

Nineteen studies included a focus on this area (Table 2). Patients reported a diverse range of symptoms^8,32,54^ with the most recent study reporting patients had an average of 10 somatic symptoms.^8^ Pain was commonly reported (prevalence ranging from 40% to 95%)^8,24,31–33,42,45,54,57^ and one study described it being worse for those with more advanced disease.^34^ Pain could be complex in nature, require multiple medications including the frequent use of opioids.^24,31,35,42,48^ Fatigue or lack of energy (prevalence ranging from 77% to 81%) and weight loss were also frequently reported.^8,45,46,54,57^ Other symptoms were wide-ranging including difficulty eating or swallowing, dry mouth, incontinence, bleeding, dyspnoea, fungating lesions, change in appetite, cough, communication difficulties, constipation, retained mucus and insomnia.^8,22,31,32,33,42,45,46,54,57,61^ One study described the intensity of nursing care needed to support patients, in part due to symptom control.^59^ Patients’ concerns about adequate symptom control were perceived as a barrier to whether or not they would be able to die at home.^52^

From the family carers’ viewpoint, pain, inability to eat and tumour fungation were reported to be the most distressing symptoms.^42^ Bereaved relatives perceived there was scope to improve on symptom management,^58^ a view shared by General Practitioners. In one study, only 45% General Practitioners perceived their patients had been satisfied with the level of symptom control.^43^

### Patient’s psychosocial and spiritual well-being

Sixteen studies included a focus on this area (Table 3). The reported prevalence of psychological distress varied.^8,21,33,44,52,55^ In the two larger studies (with more than 100 patients), more than 50% expressed psychological symptoms such as ‘worrying’, ‘fears’, ‘sadness’, or ‘depressed mood’.^8,52^ Being a burden on family carers was one specific concern raised.^51^ Within a further study, over a third (35%) of people with head and neck cancer were perceived to have some level of depression by their oncologist.^41^ Psychological symptoms manifesting as agitation or confusion were also reported during the final weeks prior to death.^28,32,58^

In terms of psychosocial support and care, one study reported that 25/40 (61%) of General Practitioners perceived their patients had received sufficient care.^42^ Perceptions from family carers varied. In one study, the ‘emotional and spiritual’ support was rated more highly (and hence needs better met) compared with management of physical symptoms.^57^ In another, however, almost 70% bereaved relatives perceived improved psychosocial support was needed during the palliative phase of illness,^43^ which led to the development of an ‘Expert Centre’ to help address these unmet needs.^50^

No study specifically focused on spiritual well-being. One qualitative study explored the ‘lived experience’ of having disfigurement and described the subsequent existential impact.^35^ A further study reported only 10/45 (23%) bereaved relatives perceived spiritual support for head and neck cancer patients had been provided.^43^

### Medical interventions in the last 12 months of life

Fourteen studies had a focus on medical interventions (Table 4). Generally, having head and neck cancer was associated with a high prevalence of ‘intensive’ interventions, especially in the last month of life. These interventions included emergency department attendance, cardio-pulmonary resuscitation, hospital admissions, intensive care admissions and ongoing chemotherapy.^25,26,31,35,37,55,64^ Factors associated with hospital admission included the presence of hypercalcaemia,^21^ respiratory or airway management issues,^31,33^ infection^35^ and problems relating to bleeding, pain and swallowing difficulties.^31^ Hospital admission could still occur frequently even when palliative care teams were involved.^64^ Two retrospective studies, conducted after death, reported that having head and neck cancer was associated with a greater risk of dying in hospital,^25,49^ although one study had very small numbers.^49^

Differing views were reported about the appropriateness of interventions. Patients could favour maximum medical interventions^52^ and bereaved relatives perceived the palliative anti-cancer treatments improved symptom management.^58^ On the other hand, however, bereaved relatives reported that treatments had been ‘too intrusive’ or not in keeping with the patients’ wishes.^44,51^

### Communication and decision-making

Fifteen papers including a focus on communication and decision-making for head and neck cancer patients (Table 5). Five papers focused more on the prevalence of the practical difficulties and issues relating to poor speech.^12,32,45,46,59^ Issues relating to communication were more common compared with other cancers,^45^ especially if the patient had a tracheostomy.^22,46,59^

Ten of the papers focused on communicating information and decision-making.^28,29,43,44,51,52,56,58,64,65^ An interview study of medical professionals in Malta, explored ethical factors influencing decision-making about treatment. The majority who were interviewed (7/10) perceived the need to provide full treatment for people with head and neck cancer, even if the prognosis was poor. Most agreed, however, that the patient formed an important part in the decision-making process.^65^ In a study assessing the communication of prognosis between healthcare professionals and patients, different ways were recognised. These included the use of numerical probability estimates, qualitative language or a combination of both.^29^ Although perceptions by patients and/or family members about the quality of communication could be good,^44,51,57,58^ there were reports of patients misunderstanding or misinterpreting information.^56^ One study described poorer levels of understanding about their illness when patients were known to palliative care teams.^64^ Within another qualitative study, dissonance between patient and family members’ information preferences was described. Patients varied between wishing for ‘full disclosure’ and a more ‘passive approach’ whereas family carers favoured being fully informed.^52^ Further communication and decision-making challenges included the information transfer between different healthcare teams^43^ and the ethical complexities that can arise relating to hydration and nutrition.^28^

### Place of death

Five studies focused on place of death, of which two were population-based^40,47^ and three were smaller cohort studies (two having less than 100 patients) (Table 6).^27,33,60^ From the population-based studies, a Taiwanese study reported over 70% head and neck cancer patients died in the acute hospital.^40^ By contrast, the other study, conducted within the UK, suggested that head and neck cancer was associated with an increased likelihood of dying in a hospice compared with other cancers.^47^

## Discussion

### Summary of main findings

Compared with other cancers, this scoping review confirms head and neck cancer patients often have complex palliative care needs, especially if there is a high degree of symptom burden. Variability in the timing and access to palliative care services, however, is recognised. Dissonance seen between patients and family carers, specifically about information needs and decision-making, are additional recognised complexities. A high prevalence of interventions such as emergency department attendance and hospital admissions occur for patients with advanced head and neck cancer even during the last weeks of life. Sole engagement with palliative care services does not necessarily negate this.

Research in this area has tended to be via single centre, quantitative studies. Few qualitative studies have been conducted with advanced head and neck cancer patients and none have focused purely on the spiritual well-being of head and neck cancer patients. There were no interventional studies identified.

### What this study adds and implications for practice and research

The key questions facing palliative care services surround the identification of who is in greatest need of referral, how these individuals should be identified, and what model of care should be provided.^66^ These questions are particularly pertinent for head and neck cancer patients who undoubtedly have palliative care needs, but for whom the method of identification and the optimum model of care provision is less clear. Our review shows variability in access to palliative care services with some patients receiving referrals late and certain groups, such as older, white, female patients, more likely to be referred.

In view of the ‘scarcity of palliative care resources,^66^ there are a number of potential ways to help identify which head and neck cancer patients would most benefit from palliative care input. Specific ‘triggers’ are recognised to help prompt palliative care consults in the emergency department, in-patient wards^67^ and from oncology services.^68^ These are generic tools aimed to screen a large population, however, rather than having been specifically validated within the head and neck cancer remit. More individualised ways to help illustrate patients concerns include the use of Holistic Needs Assessment (HNA) tools,^69^ and Patient Reported Outcome Measures, of which a vast array have been used within head and neck cancer.^70^ The Patient Concerns Inventory (PCI) is an item-prompt list specifically used to guide head and neck cancer clinical consultations including wider multi-professional engagement. Although extensively used globally, its focus of use has been with curative head and neck cancer patients, but it has potential to be adapted.^71^ Further research into which ‘screening’ tool or method would be most appropriate for initiating palliative care referral for head and neck cancer patients seems pertinent. Furthermore, clarity about how best to incorporate specific staging indicators for those recognised to be ‘high risk’^72^ in additional to individual patient needs would seem beneficial.

As well as establishing equitable referral methods, defining an appropriate model of integrated care is needed. This poses a further challenge for advanced head and neck cancer patients. Our review indicated there was a high level of interventions needed and a reliance on hospital-based care even during the last weeks of life. This finding may relate to issues arising from the use of feeding tubes and tracheostomies to help sustain vital functioning, and the fact that hospital admissions were commonly related to breathing or airway difficulties. Issues such as these can be challenging to manage in a community setting. More widely, ‘treatment related incidents’ such as those relating to other interventions, for example, urinary catheters or nasogastric tubes, are a recognised factor prompting patient safety incidents during ‘out-of-hours’ care.^72^ Additionally, obtaining timely access to care can be a challenge.^72^ Solely relying on engagement with palliative care services is not sufficient to alleviate these issues.^64^ Instead, the focus may be needed within a number of areas. Firstly, there is a need to increase integration and co-ordination between different multi-disciplinary teams to avoid ‘silo’ working patterns.^64^ Additionally, enhanced collaboration between teams^73^ would potentially help ensure timely access to specialist knowledge is more readily available. Finally, ways to help upskill healthcare professionals supporting patients within the community, and specific training about the management of tracheotomies and feeding tubes may help alleviate the need for hospital care.

Another important finding from this study was the dissonance between patient and family carer views about information needs and decision-making. Generally, unmet informational needs are recognised within advanced cancer as a whole,^15^ as well as earlier in the head and neck cancer disease trajectory.^74^ Additional complexities arise due to the many issues surrounding communication, that is inability to directly verbalise and uncertainty about the best way to communicate prognosis. Furthermore, the discussions about goals of care and the optimal ways to consider patient preferences are especially challenging in advanced disease as the treatment can be intensive and the outcomes uncertain.^16^ One national cohort study identified that 10% of people with head and neck cancer initially treated with ‘curative’ intent died within the first 12 months following diagnosis.^75^ Initiatives such as the ‘Making good decisions in collaboration’ (MAGIC) improvement programmes have been tested in early cancers involving the head and neck.^76^ An ‘Option Grids’ approach, which involves using easy-to-read decision aids with patients and healthcare professionals comparing treatment options, has also been assessed in the head and neck cancer context.^77^ Future research could focus on ways to engage advanced head and neck cancer patients and family carers more fully in shared decision-making needs, being mindful of the appropriate timing, differing information needs and the cultural sensitivity of these discussions.

In terms of research methodology, the majority of studies were quantitative observational studies conducted within single institutions. There were no interventional studies and there was only one mixed methods study, despite the unique benefits this approach can bring.^78^ Additionally, only one study explored the spiritual or existential impact of the illness. There is a real need to develop prospective multi-centre studies, using both quantitative and qualitative approaches. As well as gaining a greater understanding of needs and experiences, testing specific components of models of care would be beneficial. A broader approach to assessing holistic care, including the spiritual component, would also be important.

### Strengths and limitations

This scoping review followed an established systematic method and examined a breadth of different experiences and needs for a particularly complex subgroup of cancer patients. Our search included studies from a diverse range of countries and cultures and hence has wide-ranging relevance.

There were, however, limitations to this review. We did not conduct hand searching of key journals and grey literature was not included. Our definition of ‘advanced cancer’ was qualitatively defined rather than using specific disease staging criteria and we did not conduct additional searches using ‘place of death’ as a key search term. Additionally, we only included English language publications. In view of all these factors, some sources of data may have been overlooked. We did not focus on family carer needs per se but accept that this is an important area and would represent a focus for future study. Finally, we did not include a quality appraisal of all included studies, as within the remit of scoping reviews, risk of bias/quality appraisal is not generally recommended.^17^

## Conclusion

This scoping review has demonstrated the complexity of care for people with advanced head and neck cancer and that there are issues related to the current healthcare systems. Specific focus is needed about the optimum way those in greatest need should be identified and referred to palliative care services. Additionally, further clarity and assessment about the particular model of integrated care is required, which can address the diverse symptom needs, the communication needed to further inform decision-making, and the frequent use of interventions and issues that can arise ‘out-of-hours’ relating to these. Linkage between research and service design delivery across teams, disciplines and care settings seems key for future success.

## Supplemental Material

Supp_Table_1_Study_characteristics_REVISED_15.07.2020 – Supplemental material for The palliative care needs and experiences of people with advanced head and neck cancer: A scoping review Click here for additional data file. Supplemental material, Supp_Table_1_Study_characteristics_REVISED_15.07.2020 for The palliative care needs and experiences of people with advanced head and neck cancer: A scoping review by Catriona R Mayland, Qiaoling Marilyn Ho, Hannah C Doughty, Simon N Rogers, Prithvi Peddinti, Praytush Chada, Stephen Mason, Matthew Cooper and Paola Dey in Palliative Medicine
