# Supplementary material for: The palliative care needs and experiences of people with advanced head and neck cancer: A scoping review
Source: Palliat Med. 2020 Oct 21;35(1):27–44. doi: 10.1177/0269216320963892 (PMC7797618; doi:10.1177/0269216320963892)
Supplement: Supp_Table_1_Study_characteristics_REVISED_15.07.2020 – Supplemental material for The palliative care needs and experiences of people with advanced head and neck cancer: A scoping review [file Supp_Table_1_Study_characteristics_REVISED_15.07.2020.docx]

**Supplementary Table 1. Characteristics of all 46 studies included within the scoping review**

| **Author & year** | **Title** | **Location/setting** | **Method / population** | **Aim** | **Area of disease trajectory** | **Area(s) of needs & experiences** |
| --- | --- | --- | --- | --- | --- | --- |
| Alsirafy et al 2009^21^ | Hypercalcemia in Advanced Head and Neck Squamous Cell Carcinoma: Prevalence and Potential Impact on Palliative Care | Saudi Arabia  Single specialist hospital | Quantitative  Retrospective cohort study  Medical records of patients referred to palliative care team between Jan 2004- Dec 2007  90 HNC patients | Determine prevalence of hypercalcemia in advanced HNC patients in a palliative care setting  Assess impact of hypercalcemia on administrative data that may indicate poorer EoL care | Palliative: referral to palliative care team with evidence of ‘advanced disease’ (HNC) & measurement of serum calcium levels at least once | Medical interventions |
| Alt-Epping et al 2016^22^ | Symptoms and Needs of Head and Neck Cancer  Patients at Diagnosis of Incurability – Prevalences, Clinical Implications, and Feasibility of a Prospective Longitudinal Multicenter Cohort Study | Germany  Single hospital  (part of a nationwide multicenter  prospective longitudinal cohort study) | Quantitative  Prospective cohort study  Used questionnaires & validated tools  22 HNC patients | Assess symptoms & psychosocial needs of patients with incurable HNC | Palliative: All patients were ‘incurable’ HNC defined as inoperable local primary tumour or relapse, or metastatic disease | Physical symptoms/symptom control  Psychological & spiritual well-being  Communication &/or decision-making |
| Becker et al 2011^23^ | Palliative Cancer Care: An Epidemiologic Study | Germany  Single large university medical centre (hospital) | Quantitative  Prospective cohort study  Electronic medical records review of 10,679 patients between Jan 2004- May 2005 (477 HNC patients) | Identify  percentage of cancer patients with palliative care needs (PCN) | Palliative: All patients deemed to have ‘palliative care needs’ using WHO definition & assessed by treating physician | Overall palliative care needs & access to services |
| Bisht et al 2011^24^ | Effect of Palliative Drug Therapy on Quality of life in Advanced Head and Neck Cancer Patients | Himalayas, India  Single medical institute (tertiary care centre) | Quantitative  Prospective cohort study  Used own QoL survey consisting of VAS  40 HNC patients | Examine effect of palliative drug therapy on QoL in advanced HNC patients over 2 month period | Palliative: All patients either had stage IV HNC, recurrent cancer or distant metastasis | Physical symptoms/symptom control |
| Chang et al 2015^25^ | Determinants for Aggressive End-of-Life Care for Oral  Cancer Patients | Taiwan  Population-based study | Quantitative  Retrospective cohort study  Used linked databases of 5386 patients who died from oral cancer between 2009-2011 | Investigating relationship between demographics, primary physician’s specialty, hospital characteristic & ‘aggressiveness’ of their EoL care | Post-death: Died from oral cancer (using national death registry data)  3445 (65%) had metastatic disease | Medical interventions |
| Chen et al 2009^26^ | A population-based study on the prevalence and determinants of cardiopulmonary resuscitation (CPR) in the last month of life for Taiwanese cancer decedents, 2001–2006 | Taiwan  Population-based study | Quantitative  Retrospective cohort study  Used linked databases of 204,850 patients dying between 2001-2006  17,040 HNC patients | Determine prevalence of CPR in Taiwanese cancer patients in the last month of life & association with patient & physical characteristics | Post-death: Died from cancer (using national death registry data) | Medical interventions |
| Chen et al 2014^27^ | Determinants of Preference for Home Death Among Terminally Ill Patients With Cancer in Taiwan: A Cross-Sectional Survey Study | Taiwan  National-wide sample from oncology units within 24 hospitals | Quantitative  Cross-sectional survey (convenience sample)  2188/3125 (70% response rate)  Terminally ill cancer patients not under hospice care between Feb 2003-Nov 2004  111 HNC patients | Determine impact of patient demographics, disease characteristics, prognosis awareness & support network variables on preference for home death | Palliative: Had malignancy diagnosed by primary physician as terminal stage, continuing to progress & unresponsive to curative treatments | Place of death |
| Chiu et al 2000^28^ | Ethical dilemmas in palliative care: a study in Taiwan | Taiwan  Single palliative care unit within a hospital | Quantitative  Prospective cohort study  HCP documentation of ethical dilemmas from 246 patients between July 1997 & June 1998  16 HNC patients (6.5%) | Identify most frequently encountered ethical dilemmas in the palliative care unit | Palliative: All patients with a duration of stay in palliative care unit of > 2 days | Communication &/or decision-making |
| Dronkers et al 2018^29^ | Communication of prognosis in head and neck cancer patients; a descriptive  qualitative analysis | Holland  Out-patient clinic in single cancer centre institute | Qualitative  Descriptive study using audio-taped physician-patient consultations in which treatment options were discussed  23 patients & 7 HNC surgeons  3 patients had palliative disease | Investigate whether prognostic information on life expectancy is included during communication on diagnosis & treatment plans between physicians and HNC patients in all phases of illness  Describe communication style displayed by physicians | Curative and palliative: Patients receiving a treatment proposal for their recently diagnosed H&N cancer | Communication &/or decision-making |
| Enomoto et al 2015^30^ | The cost of hospice services in terminally ill patients with head and neck cancer | USA  Nationwide hospitals using Surveillance, Epidemiology, and End Results (SEER) database | Quantitative  Retrospective cohort study  Database review of SEER – Medicare linked system between Jan 1995-Dec 2007  7383 HNC patients | Compare monthly Medicare costs for all services used during last 12 months of life by HNC patients comparing those who received hospice care with those who did not | Post-death: Died from oral or pharyngeal cancer | Overall palliative care needs & access to services |
| Ethunandan et al 2005^31^ | Quality of dying in head and neck cancer patients: A retrospective analysis  of potential indicators of care | UK  Single hospital | Quantitative  Retrospective cohort study  Medical chart review of 32 randomly selected HNC patients who died between Aug 1999-Dec 2002 | Evaluate quality of dying experience by examining symptoms in the last week of life | Post-death: Died from HNC  Focus on last week of life | Psychological & spiritual well-being  Physical symptoms/symptom control  Medical interventions |
| Forbes 1997^32^ | Palliative care in patients with cancer of the head and neck | UK  Single hospice | Quantitative  Retrospective cohort study  Medical chart review of 38 patients with HNC admitted to hospice over 3-year period from 1990 | Outline nature, incidence & management of problems & the role of the hospice in the patients care | Palliative: patients with HNC diagnosis admitted to hospice | Physical symptoms/symptom control  Communication &/or decision-making |
| Fullarton et al 2016^33^ | Analysis of deaths between 2007 and 2012 of patients with head and neck cancer on a surgical ward at a regional centre or in an independent hospice | UK  Surgical ward in regional HNC centre & hospice on same hospital site | Quantitative  Retrospective cohort study    Electronic medical chart review of 76 HNC patients who died between 2007 & 2012 | Record characteristics, mode of death & potential indicators of the quality of care at the end of life for HNC patients | Curative and palliative: died on surgical ward or in hospice | Psychological & spiritual well-being  Physical symptoms/symptom control  Medical interventions  Place of death |
| Gupta et al 2017^34^ | Predictors affecting quality of life in patients with upper  aerodigestive tract cancers: a case-control study from India | India  Two hospitals | Quantitative  Case-control study (n=480)  240 cases: hospital patients 30-80 years; recruited between June 2014-May 2015; Diagnosed with upper aerodigestive tract cancer (UADT)  240 controls: randomly selected, diagnosed with disease other than UADT cancer; in-patient or out-patient setting; matched for gender & age | Measure QoL in UADT cancer patients in comparison with hospital controls  Assess impact of clinical predictors at time of diagnosis on QoL (using the University of Washington QoL questionnaire) | Curative and palliative (stages I-IV)  26/240 had stage IV disease; 29/240 had stage III disease | Psychological & spiritual well-being  Physical symptoms/symptom control |
| Heinonen et al 2018^35^ | End-of-life care pathway of head and neck cancer patients: single institution  experience | Finland  Single university hospital | Quantitative  Retrospective review  Hospital charts of 60 HNC patients | Describe current status of palliative care of HNC patients in one specific university hospital region | Palliative: defined as the disease period when curative or life-prolonging treatment can no longer be offered | Physical symptoms/symptom control  Medical interventions |
| Henry et al 2014^36^ | Looking beyond Disﬁgurement: The experience of patients with head and neck cancer | Canada  HNC surgical out-patient clinics from two hospitals | Qualitative  Interviews with 14 HNC patients who had undergone disfiguring surgery | Understand lived experience of disfigurement in HNC & explore what patients considered to be its influences | Curative and palliative: 9/14 had ‘advanced disease’ (stage III or IV) | Psychological & spiritual well-being |
| Henson et al 2018^37^ | What factors influence emergency department visits by patients with  cancer at the end of life? Analysis of a 124,030 patient cohort | England  Population-based study | Quantitative  Retrospective cohort study  Electronic databases of 124,030 cancer patients who died between April 2011-March 2012  2789 HNC patients | Identify socio-demographic and clinical factors associated with end-of-life emergency department (ED) visits  Determine relationship between patients’ prior emergency department use and risk of multiple visits (>=2) in last month of life | Post-death: Died from cancer (using national death registry data) | Medical interventions |
| Hui et al 2012^38^ | Access to palliative care among patients treated at a comprehensive cancer center | USA  Single cancer centre | Quantitative  Retrospective cohort study  Medical record review of 816 cancer patients who died between Sept 2009-Feb 2010  48 HNC patients | Determine proportion & predictors of cancer patients who receive palliative care (PC) | Post-death: Died from advanced cancer (defined as locally advanced, metastatic, or recurrent disease; incurable disease at presentation; refused all curative treatments; referred to phase I trials; oncologists deemed incurable e.g low performance status, comorbidities) | Overall palliative care needs & access to services |
| Johnstone et al 1998^39^ | Identifying potential need for cancer palliation in Nova Scotia | Canada  Population-based within specific region | Quantitative  Retrospective cohort study  Review of electronic health data for all adults in Nova Scotia (n=14494) who died of cancer from 1988-1994  44 HNC patients | Assess whether Nova Scotia cancer patients (who may need palliative care) are being referred to the comprehensive Halifax-based Palliative Care Program (PCP) | Post-death: Died from cancer (obtained from Cancer registry database) | Overall palliative care needs & access to services |
| Kuo et al 2017^40^ | End-of-life care for head and neck cancer patients: a population-based study | Taiwan  Population-based | Quantitative  Retrospective cohort study  Review of electronic databases for 98,221 patients diagnosed with HNC between Jan 1997 & Dec 2010 | Assess end-of-life care for patients with HNCs in Taiwan | Post-death: Patients diagnosed with HNC who subsequently died between January 2005 - December 2011 | Place of death |
| Kwon et al 2013^41^ | Clinical Characteristics of Cancer Patients Referred Early to Supportive and Palliative Care | USA  Single supportive care centre (SCC) | Quantitative  Retrospective cohort study  Electronic medical records review of 200 consecutive cancer outpatients referred to the SCC between Aug 2008 - Oct 2010  73 (37%) HNC patients | Define characteristics, outcomes & utilization of medical services by cancer patients referred early in their disease course to outpatient palliative care services | Curative and palliative: defined as having a diagnosis of malignancy | Overall palliative care needs & access to services |
| Lal et al 2016^42^ | Initial experience of head and neck cancer patients treated in an oncologist led palliative cancer care clinic at a tertiary cancer centre in Uttar Pradesh: is the initiative a of a full-fledged palliative care for cancer patients justified | India  Single palliative cancer care clinic attached to oncology unit | Quantitative  Retrospective cohort study  Medical record review of 153 HNC patients  Supplementary information provided by single telephone consult with 63 NOK after death | Evaluate range of symptoms, other needs & evaluation of treatment strategies, especially for pain management | Palliative: advanced HNC treated radically & subsequently failed on treatment or late presentation (all stage III or IV disease) | Psychological & spiritual well-being  Physical symptoms/symptom control |
| Ledeboer et al 2006^43^ | Palliative care for head and neck cancer patients in general practice | Holland  Community setting linked with single large university medical centre | Quantitative  Retrospective cross-sectional postal questionnaire  55 GPs caring for HNC patients who died between Jan 2003- July 2004  41 GPs completed (75% response rate) | Evaluate experience of GPs in the care of palliative HNC patients, experiences of communication & consultation of attending specialists | Palliative: incurable and died from HNC | Psychological & spiritual well-being  Physical symptoms/symptom control  Communication &/or decision-making |
| Ledeboer et al 2008^44^ | Experience of palliative care for patients with head and neck cancer through the eyes of next of kin | Holland  Single large university medical centre | Quantitative  Retrospective cross-sectional postal questionnaire  55 bereaved relatives/close friends to those with incurable HNC  45 completed (82% response rate) | Increase knowledge of how treatment & support are experienced by relatives of palliative HNC patients during the palliative stage & after death | Palliative: incurable HNC | Psychological & spiritual well-being  Medical interventions  Communication &/or decision-making |
| Lidstone et al 2003^45^ | Symptoms and concerns amongst cancer outpatients:  identifying the need for specialist palliative care | UK  Out-patient clinics in large single cancer centre | Quantitative  Prospective cross-sectional survey  480 patients from 8 different tumour groups  60 HNC patients - 30% advanced (non-curative intent) | Assess prevalence & severity of symptoms & concerns - identify patient groups who might benefit from routine SPC involvement in outpatient clinics | Curative and palliative: histologically proved diagnosis of cancer | Psychological & spiritual well-being  Physical symptoms/symptom control  Communication &/or decision-making |
| Lin et al 2011^46^ | Symptom Patterns of Patients with Head  and Neck Cancer in a Palliative Care Unit | Taiwan  Single palliative care unit within a hospital | Quantitative  Retrospective cohort study  Medical chart review of 94 terminal HNC patients admitted to palliative care unit between May 2006- Dec 2008 | Describe symptom patterns of terminal HNC patients in palliative care unit | Palliative: ‘terminal’ HNC admitted to palliative care unit who subsequently died | Physical symptoms/symptom control  Communication &/or decision-making |
| Lock and Higginson 2005^47^ | Patterns and predictors of place of cancer death for the oldest old | UK  Population-based study | Quantitative  Retrospective cohort study  Cross-sectional database analysis of 315,462 people  aged 75 and over dying of cancer in England and Wales between 1995-1999 | Describes the older population who die of cancer & the factors which may affect  place of death | Post-death: Died from cancer from national death registry data | Place of death |
| Lokker et al 2013^8^ | Symptoms of patients with incurable head and neck cancer: Prevalence and impact on daily functioning | Holland  Single university medical centre | Quantitative  Cross-sectional descriptive study (with retrospective & prospective components) with 124 HNC patients  Additional symptom impact questionnaire with convenience sample of 24 patient/family carer dyads | Determine prevalence & impact of symptoms on daily functioning in HNC patients during palliative phase  Examine discrepancies between patients & family members symptom scoring | Palliative: primary head and neck tumour in the ‘palliative phase’ | Psychological & spiritual well-being  Physical symptoms/symptom control |
| Mercadante 1998^48^ | Opioid responsiveness in patients with  advanced head and neck cancer | Italy  Single community setting | Quantitative  Prospective cohort study  Review of 37/107 advanced HNC patients who required opioid therapy >6 weeks between Jan 1994-Sept 1997 | Establish degree of opioid sensitivity & possible factors involved in advanced stage HNC patients being followed up at home | Palliative: advanced HNC stages 3–4 | Physical symptoms/symptom control |
| Mercandante et al 2016^49^ | The characteristics of advanced cancer patients followed at home, but admitted to the hospital for the last days of life | Italy  Single community setting within specific region | Quantitative  Retrospective cohort study  Medical chart review 550 patients followed by a home care program between Aug 2009-Dec 2013  20 HNC patients | Assess patient characteristics who were hospitalized in last days of life after being assisted by a home palliative care team; identify possible risk factors for hospitalization. | Palliative: Those with advanced cancer known to home care program | Medical interventions |
| Mulvey et al 2016^50^ | Use of inpatient palliative care services in patients with metastatic incurable head  and neck cancer | USA  Population-based study | Quantitative  Retrospective cohort study  Database review between 2001 & 2010 of 80,500 adult HNC inpatients | Determine incidence of palliative care consultations (PCC) among hospitalized metastatic, incurable HNC patients  Examine relationship between palliative care encounters & in-hospital morbidity, mortality, length of hospitalization & costs | Palliative: All patients had diagnosis of a metastatic malignant oral cavity, laryngeal, hypopharyngeal, or oropharyngeal cancer | Overall palliative care needs & access to services |
| Offerman et al 2014^51^ | Experience of palliative care for patients with head and neck cancer  through the eyes of next of kin: Impact of an expert center | Holland  Single medical centre | Quantitative  Retrospective cross-sectional survey  40 bereaved relatives to HNC patients & comparing results to  similar group of HNC patients | Evaluate interventions/impact of newly established ‘Expert Center’ on palliative HNC patients as perceived by bereaved relatives | Palliative: patients had incurable HNC | Psychological & spiritual well-being  Medical interventions  Communication &/or decision-making |
| O’Sullivan & Higginson, 2016^52^ | ‘I’ll continue as long as I can, and die when I can’t help it’: a qualitative exploration of the views of end-of -life care by those affected by head and neck cancer | Ireland  Single Regional Ear, Nose and Throat (ENT) Department | Qualitative  Interviews with 7 HNC patients & 3 family carers | Explore Irish HNC patient & care-givers views on EoL care | Curative and palliative: 3 patients with stage III or IV disease | Psychological & spiritual well-being  Physical symptoms/symptom control  Medical interventions  Communication &/or decision-making |
| Patil et al 2018^53^ | Distress Management in Patients With  Head and Neck Cancer Before Start of  Palliative Chemotherapy: A Practical  Approach | India  Single oncology department within medical centre/hospital | Quantitative  Prospective cohort study  Single arm observational study of 200 HNC patients enrolled between Dec 2015- April 2016 prior to palliative chemotherapy | Identify the incidence of distress in HNC patients undergoing palliative chemotherapy & the factors associated with it | Palliative: all HNC patients undergoing palliative chemotherapy | Psychological & spiritual well-being |
| Price et al 2009^54^ | Symptoms and Terminal Course of Patients Who Died of Head and Neck Cancer | USA  Single cancer centre | Quantitative  Retrospective cohort study  Medical chart review of 93 HNC patients who died between 1999 & 2001 | Understand cause & location of death & symptoms experienced at the end of life | Palliative: all patients died from HNC | Physical symptoms/symptom control |
| Randen et al 2013^55^ | Treatment decisions and discontinuation of palliative chemotherapy near the end-of-life, in relation to socioeconomic variables | Sweden  Single oncology institute | Quantitative  Retrospective cohort study  Electronic medical record review of 346 cancer patients dying between April-Nov 2009  25 HNC patients | Describe how palliative chemotherapy is prescribed at the end-of-life to patients | Palliative: All cancer patients dying from disseminated disease | Medical interventions |
| Roscoe et al 2013^56^ | Beyond Good Intentions and Patient Perceptions: Competing Definitions of Effective Communication in Head and Neck Cancer Care at the End of Life | USA  Single specialised clinic in a single tertiary cancer centre | Quantitative  Prospective study  Cross-sectional structured interviews with 14 HNC patients & 8 healthcare providers | Understand ways in which end-stage HNC patients & their oncologists talk about end-of-life issues | Palliative: defined as newly diagnosed, persistent or recurrent cancer; who had been told by oncologist that prognosis was ‘terminal’ (likely < 6 months) | Psychological & spiritual well-being  Physical symptoms/symptom control  Communication &/or decision-making |
| Rylands et al 2016^57^ | Outcomes by area of residence deprivation in a cohort of oral cancer patients: survival, health-related quality of life and place of death | UK  Single university hospital | Quantitative  Retrospective cohort study  Electronic case record review of 553 HNC patients between 2008-2012  69 palliative patients (12%)  Included results from completed University of Washington QoL questionnaire & calculated IMD scores | Report treatment selection, survival, health-related quality of life, cause & place of death in relation to deprivation status | Curative and palliative: treated for SCC HNC | Overall palliative care needs & access to services |
| Schuman et al 2011^58^ | End-of-Life Care among Head and Neck Cancer Patients | USA  Single tertiary cancer centre | Quantitative  Cross-sectional survey  Telephone survey using validated questionnaire to family members of 371 deceased HNC patients  58 participants (20% response rate) | Determine perceived quality of care for HNC patients at the end of their lives | Palliative: focus on the last week of life | Psychological & spiritual well-being  Physical symptoms/symptom control  Medical interventions  Communication &/or decision-making |
| Sesterhenn et al 2008^59^ | End-of-Life Care for Terminal Head  and Neck Cancer Patients | Germany  Single HNC department within university hospital | Mixed methods  Retrospective medical chart review of 16 HNC patients who were transferred to hospice  Interviews with hospice staff following ‘standard protocol’ | Describe end-stage disease in advanced HNC patients – circumstances of final period of life & describe period in hospice setting | Palliative: ‘end-stage’ disease who were transferred to the hospice | Psychological & spiritual well-being  Physical symptoms/symptom control  Communication &/or decision-making |
| Shah D et al 2017^60^ | Outcome of Head and Neck Cancer Patients Who Did Not Receive Curative-Intent Treatment | Australia  Single hospital MDT | Quantitative  Retrospective cohort study  Clinical records review of 412 HNC patients who had undergone MDT assessment between Jan 2013 - Dec 2014  74 (18%) palliative HNC | Estimate frequency of referral of HNC patients to ‘terminal care’  Ascertain where & when the patient died | Palliative: MDT decision that patient had incurable cancer (either due to staging, co-morbidities or patient choice) | Overall palliative care needs & access to services  Place of death |
| Shinozaki T et al 2017^61^ | Quality of life and functional status of terminally ill head and neck cancer patients: a nation-wide, prospective observational study at tertiary cancer centers in Japan | Japan  Nation-wide study of 11 cancer centres & university hospitals | Quantitative  Prospective cross-sectional cohort study  Involving 100 in-patients with HNC | Examine relationship between QoL & functional status in terminally ill HNC patients  (using the European Organization for Research  and Treatment of Cancer Quality of Life Questionnaire (EORTC  QLQ)-Core 15-Palliative Care (C15-PAL) | Palliative: diagnosis of incurable HNC | Physical symptoms/symptom control |
| Tang et al 2010^62^ | A population-based study on the determinants of hospice utilization in the last year of life for Taiwanese cancer decedents, 2001-2006 | Taiwan  Population based | Quantitative  Retrospective cohort study  Review of electronic administrative data among 204,850 Taiwanese cancer decedents, 2001–2006  17040 HNC patients | Evaluate associations between hospice utilization in the last year of life & patient demographics, disease characteristics, physician specialty, hospital characteristics, & availability of healthcare resources at the hospital and regional levels in Taiwan | Died from cancer (using national death registry data) | Overall palliative care needs & access to services |
| Timon & Reilly 2006^63^ | Head and neck mucosal squamous cell carcinoma: results of palliative management | Ireland  Single consultant surgeons’ practice | Quantitative  Retrospective cohort study  Chart review & database extraction of 60/286 SCC HNC patients referred to a single HNC surgeon between July 1994- July 1999 | Assess group of incurable HNC patients presenting for the first time to one surgeon  Emphasis on natural history & palliative therapy required | Palliative: Treatment plan at diagnosis was non-curative/palliative | Overall palliative care needs & access to services |
| Ullgren et al 2017^64^ | Working in silos? Head and neck cancer patients during and after treatment with and without early palliative care referral | Sweden  Specific community region (Stockholm-Gotland) | Quantitative    Cross-sectional questionnaire  203/289 adult HNC patients (70% response rate)  43 (21%) been referred to palliative care services | Describe HNC patients referred to palliative care & how care transition from acute oncological to palliative care impacted on Health Related Quality of Life (HRQoL) & information  Explore HNC patients' HRQoL & perceived information | Curative and palliative: all adult HNC patients – comparison between those referred to palliative care services and those who hadn’t been | Medical interventions  Communication and decision-making  Overall palliative care needs & access to services |
| Xuereb et al 2015^65^ | Local attitudes in the treatment of low prognosis head and neck squamous cell carcinoma | Malta  National study involving hospital healthcare professionals | Qualitative  Interviews with 10 medical healthcare professionals | Explore local decision-making, from an ethical point of view, about HNC | Curative and palliative: ‘low prognosis HNC’ | Communication &/or decision-making |

Abbreviations: EoL = end of life; GPs = General Practitioners; HNC = head and neck cancer; IMD = Index of multiple deprivation; MDT = multi-disciplinary team; NOK = next-of-kin; QoL = quality of life; VAS = visual analogue scale; WHO = World Health Organisation
